# Identification of Prognostic and Predictive Osteosarcoma Biomarkers

**DOI:** 10.3390/medsci7020028

**Published:** 2019-02-11

**Authors:** Radoslav Zamborsky, Milan Kokavec, Stefan Harsanyi, Lubos Danisovic

**Affiliations:** 1 Department of Orthopedics, Faculty of Medicine, Comenius University, Limbova 1, 833 40 Bratislava, Slovakia; kokavecm@hotmail.com; 2 Institute of Medical Biology, Genetics and Clinical Genetics, Faculty of Medicine, Comenius University, Sasinkova 4, 811 08 Bratislava, Slovakia; stefan.harsanyi@fmed.uniba.sk (S.H.); lubos.danisovic@fmed.uniba.sk (L.D.)

**Keywords:** osteosarcoma, chemotherapy, treatment, malignant bone tumors, biomarkers

## Abstract

Both adolescents and children suffer from osteosarcoma, localized in the metaphysis of the long bones. This is the most common primary high-grade bone tumor in this patient group. Early tumor detection is the key to ensuring effective treatment. Improved osteosarcoma outcomes in clinical trials have been contingent on biomarker discovery and an evolving understanding of molecules and their complex interactions. In this review, we present a short overview of biomarkers for osteosarcoma, and highlight advances in osteosarcoma-related biomarker research. Many studies show that several biomarkers undergo critical changes with osteosarcoma progression. Growing knowledge about osteosarcoma-related markers is expected to positively impact the development of therapeutics for osteosarcoma, and ultimately of clinical care. It has also become important to develop new biomarkers, which can identify vulnerable patients who should be treated with more intensive and aggressive therapy after diagnosis.

## 1. Introduction

The term osteosarcoma, i.e., disease of bones, was first mentioned in the “Lectures of Boyer” in 1807 [1]. Approximately 8.7 per million in children and adolescents under the age of 20 years suffer from osteosarcoma (OS) [2], which originates from mesenchymal tissues. This makes it the most frequent malignant tumor of the bone in the pediatric age group [3]. The hallmark symptoms of OS include pain, limited joint movement, and swelling of the affected area. The diagnosis of OS is based on a set of clinical evaluations, radiological examination and histological evaluation of biopsy. Histological evaluation provides definitive diagnosis and also information about the tumor grade (Table 1).

Five-year survival post diagnosis is higher in patients with no metastases in comparison to patients with metastases [4]. During the growth spurt, the incidence of OS peaks and is commonly seen in the metaphyseal region of long bones; the distal femur (43%), proximal tibia (23%) and proximal humerus (10%) [5]. The majority of metastases occur in the lungs [6]. The incidence of the disease is higher in males than in females [7]. Only 10% of patients survived before 1970. Neoadjuvant and adjuvant chemotherapy and surgical resection have improved the survival rate to 50–70% [5]. Despite these efforts, the metastatic OS survival rate is still 10–20% [8] and has changed little in the last three decades. The main drawback for effective treatment is the heterogeneous nature of this malignancy.

Many prognostic factors have been researched for OS (Table 2), but they have not improved the outcomes of this disease. This may be due to the studies being flawed, too heterogeneous or lacking valid assessments [9]. The identification of biomarkers for diagnosis and prognosis of OS patients, especially in patients with metastases may prove a much-needed tool for early diagnosis of the disease, as well as to identify potential therapeutic targets. Serum biomarkers have been used for the prognosis of other cancers but few have been characterized in OS.

This review focuses on some of the markers that have been studied in the context of OS, in cell lines, in animal models, and in OS patients.

## 2. MicroRNAs

By definition, microRNAs, or miRNAs, are highly conserved, endogenous, tightly regulated, small (18–25 nucleotide), non-coding RNAs, that target and inhibit mRNAs by direct degradation or by translational inhibition. The biological roles of miRNAs include processes such as cell death differentiation and cell cycle. The expression of miRNAs is tightly regulated by epigenetic mechanisms such as DNA methylation, histone deacetylation, and other transcriptional regulation mechanisms.

Owing to their inhibitory functions, miRNAs can inhibit oncogenes or tumor suppressor genes, which suggests an important role for these molecules in oncogenesis. The first such study on the involvement of miRNA in cancer was by Calin et al., where they showed that the genes miR15 and miR16 were either deleted or downregulated in chronic lymphocytic leukemia [10]. Since then, it has been found that miRNAs are dysfunctional in other types of cancers such as breast cancer, colon cancer, and lung cancer, as well as OS, whereby some miRNAs overexpressed in certain types of cancer while others are underexpressed. In the case of OS, various studies reported the expression profile of different miRNA in either cell lines or in OS samples [11,12,13,14]. Some examples of miRNAs that are upregulated in OS are miR-195, miR-99, miR-181, and miR-148a, while others such as miR-539 miR-145 and miR-335 were found to be downregulated in MG-63 human OS cell lines. An elegant review by Zhang et al. discusses in detail, the role of miRNAs in OS [15]. These observations have led to the hypothesis that miRNAs could potentially be used as biomarkers for the diagnosis and prognosis of OS. In this direction, many basic, translational and clinical studies have been carried out in the past decade that have investigated levels of different miRNAs in OS samples with in vivo or in vitro studies. One such study on the role of miRNA as a marker of OS was carried out by Cai et al., where they showed that levels of miR-195, as determined by real-time quantitative reverse transcription polymerase chain reaction (RT-PCR), was lower in the serum of patients with OS when compared to normal controls, suggesting a potential role of miRNA as a serum marker for diagnosis of OS [16]. This evidence on miRNAs shows that it can be effectively used for OS detection and monitoring. The quantitative RT-PCR assay was used to show that serum miR-199a-5p concentrations were higher in OS samples [17]. Differentially expressed genes (DEG) were investigated on OS samples [18]. The authors found that out of 323 DEGs, 134 were upregulated and 189 downregulated. The increased expression of DEGs was associated mainly with a reorganization of the cytoskeleton. On the other hand, DEG downregulation plays pivotal role in wound healing. Two miRNAs and their coding genes (miR-202 and miR-9) were also identified in this study. Martand colleagues focused their investigation on a selected category of genes [19]. They analyzed gene expression in patients with sporadic pediatric OS by digital expression profiling. They found that RUNX2, CDC5L, MDM2, RECQL4, and CDK4 are associated with OS and may be used as reliable predictive biomarkers of differential response to chemotherapy. In the next study, Ouyang et al. measured the expression levels of six miRNAs (miR-21, miR-199a-3p, miR-143, miR-34, miR-140, and miR-132) and showed that the miR-21 level was higher in OS patients, whereas miR-199a-3p and miR-143 were decreased in OS patients [20].

Knowledge about differential levels of miRNA has greatly enhanced our understanding of the disease. miRNAs are one of the most characterized biomarkers investigated in the pathophysiology of OS and are promising candidates for the prognosis and diagnosis of OS. However, much work is needed to translate this information from benchside to bedside, requiring randomized clinical trials and the discovery of drugs to modulate levels of miRNAs associated with OS in the future, which could prove more difficult than originally thought.

## 3. Immune Markers

Cytokines are small proteins that are secreted by cells such as monocytes and macrophages. Cytokines are regulators of host responses to infection, immune responses, inflammation, and trauma. An abnormal increase in plasma levels of proinflammatory cytokines is associated with obesity, type 2 diabetes, atherosclerosis, rheumatoid arthritis and cancer. Several groups tested the presence or absence of cytokines in OS. Levels of transforming growth factor beta (TGF-β) were found to be elevated in OS patients when compared to healthy controls [21]. Pro-angiogenic effects of TGF-β on OS have also been reported [22]. Drugs that inhibit TGF-β have been used in preclinical studies and in clinical trials [23]. Zhu and colleagues studied the levels of cytokines in OS patients by using antibody microarray assay [24]. Interestingly, they found that twenty-one cytokines including interleukin-6, protein-1, tumor growth factor-β, growth-related oncogene, hepatocyte growth factor, chemokine ligand 16, endoglin, matrix metalloproteinase-9 and platelet-derived growth factor-AA were upregulated in OS samples compared with control samples and this was later confirmed by performing enzyme-linked immuno sorbent assay (ELISA). In another study, β-isomerized C-terminal telopeptides (β-CTx) and total pro-collagen type 1 amino-terminal propeptide (tP1NP) were found to be significantly higher in OS patients [25], as measured by immunoassay on the serum of volunteers. Thus, levels of these proteins can also be used as OS biomarkers. Mesenchymal stem cells are known to secrete IL-6 which promotes tumor proliferation and metastasis [26,27]. A study by Tu et al. [28] determined effects of OS cells on the production of pro-tumor cytokines by mesenchymal stem cells (MSCs). Cultured medium from cell lines Saos-2 and U2-OS stimulated the production of IL-6 and vascular endothelial growth factor (VEGF) in MSCs; theantibody to TGF-β neutralized this effect.

TIM3, belongs to the family of T cell Ig- and mucin-domain-containing molecules (TIMs). TIM3 is expressed by many immune cells including T cells, macrophages, dendritic cells, and natural killer cells [29]. Abnormal TIM3 expression has been associated with carcinomas [30,31]. Shang and colleagues found TIM3 to be expressed in nine invasive human OSs [32]. Moreover, TIM3 was found to be co-expressed with Slug, Snail and Smad, which have been detected in OS patients [33,34]. Thus, TIM3 is yet another promising candidate as a diagnostic and therapeutic tool for OS.

IgM antibodies. Tumor-specific antibodies can be detected in very early stages which makes them suitable candidate biomarkers. In the category of natural antibodies against tumors, IgM isotypes are most prominent. IgM antibodies secreted by B1 cells are the first spontaneous antibodies produced against foreign and self-antigens despite the lack of previous exposure. This property of IgM antibodies allows for an immediate protective immune response against invading pathogens. IgM activity against tumor-specific antigen ANG has been detected in OS. In a study using ELISA to analyze the presence of ANG–IgM immune complexes in the sera of patients with OS, serum ANG–IgM levels were found to be significantly higher in OS patients when compared to healthy individuals [35].

Matrix metalloproteinases (MMPs) belong to a family of zinc-dependent endopeptidases. MMPs are secreted by macrophages and neutrophils and are involved in processes of extracellular matrix (ECM) degradation and remodeling. MMPs participate in many physiological and pathological processes, including morphogenesis, wound healing, tissue repair, and remodeling. Moreover, MMPs play a crucial role in tumor progression through the activation of cell growth, migration, invasion, metastasis, and angiogenesis. Matrix metalloproteinase 9 (MMP-9) is overexpressed in various tumors and related to poor disease prognosis in oral and gastric cancers. A meta-analysis revealed that MMP-9 expression was associated with increased risk of patients with OS. This study also found that there was a significant correlation between MMP-9 expression and OS risk among the Asian and non-Asian population [36]. Liu et al. examined the possible prognostic value of MMP-9 in OS. They used meta-analysis and concluded that MMP-9 expression and survival of patients with OS correlates with an adverse prognosis for OS [37]. However, this study was challenged by Zhang and colleagues, and thus more studies are needed to analyze the significance of MMP9 as a biomarker in OS [38].

Galectins are beta-galactoside binding proteins of the lectin family and are implicated in cellular processes such as migration [39], apoptosis, and differentiation [40,41]. Among the members of this family, galectin 1 and 3 have been implicated in cancers such pancreatic cancer, breast cancer, and gastric cancer as well as OS [42]. Galectin 1 and 3 have been shown to have a positive correlation with OS. In a study by Zhou et al., the serum levels of galectin 3 were found to be higher in OS patients compared to healthy controls and increasingly higher levels of the protein were observed in advanced stages [43]. Similarly, in a study by Lei et al., galectin 3 was found to be overexpressed in OS tissues and OS cell lines. Knockdown of galectin 3 led to inhibition of growth, migration, and invasion of OS cell lines [44]. Similar results were reported by another group who demonstrated that silencing of galectin 3 led to decreased migration and invasion [45] and this correlated with reduced expression of mediators of invasion such as Lyn, beta-catenin and Src. Similar to galectin 3, knockdown of galectin 1 was also found to inhibit migration and invasion properties of OS cells and this was found to be dependent on mitogen-activated protein kinase pathway [46]. Galectin 1 is also a diagnostic marker for differentiating between chondroblastic OSs and conventional chondrosarcoma [47]. These findings suggest roles for galectin 1 and 3 as biomarkers of OS and that their expression levels may be correlated with the progression of the disease.

## 4. Other Protein Markers

In order to survive in their hypoxic environment, cancer cells activate their hypoxia-inducible transcription factor (HIF-1), which consists of alpha and beta subunits. HIFα is overexpressed in many cancers such as lung, colon, breast, prostate, and skin cancers [48]. Similar to HIF α, VEGF, a hypoxia-responsive factor has also been observed to be upregulated in many types of cancers. VEGF is involved in the tumor angiogenesis, and its expression has been used as a prognostic biomarker in patients with OS [49]. Moreover, VEGF expression is also associated with both the overall survival rate and the disease-free survival rate. Compared with OS patients with low or negative VEGF expression, high VEGF expression in patients was associated with lower disease-free survival and lower overall survival.

Both HIF and VEGF have also been found to be upregulated in the OS cell line in response to hypoxia [50]. Mizobuchi et al. [51] reported that metastases were present in 61% of HIF-1α-positive OS patients, representing a 4.3-fold greater risk for having metastatic disease in patients with HIF-1α expression compared to that of HIF-1α-negative patients. In addition, increased VEGF protein expression was observed more frequently in OS female patients than in male patients. A fairly recent meta-analysis study that looked at the HIFα expression in OS patients, found that high HIF-1 expression was associated with a worse prognosis when compared to low or undetectable HIF-1 expression.

In another study analyzing the role of VEGF in OS prognosis, higher VEGF expression was associated with lower disease-free and lower overall survival in patients. This suggests a role for VEGF as an effective biomarker for the prognosis of OS [52]. However, further studies with larger sample sizes are needed to validate these findings.

Another marker that may have a role in prognosis is apurinic/apyrimidinic endonuclease 1 (APE1), which is involved in DNA base excision repair pathway. APE1 also plays a role in apoptosis, and cell cycle, and regulates other transcription factors such as HIF alpha, nuclear factor kappa B (NF-κB), and tumor-suppressor protein p53 [53]. Changes in levels of APE1 have been found in various types of cancers such as lung, ovarian, and prostate cancers. Altered expression of APE1 is also found in OS, with high levels of APE1 correlated with reduced survival times among patients with OS. Knocking down expression of APE1 using siRNA led to enhanced cell sensitization to the DNA damaging agents [54].

Hypoxia also leads to the activation of cyclooxygenase-2 (COX-2) expression. COX2 overexpression has been related to tumor development and it has been shown that COX-2 promotes the pathogenesis of cancer [55,56,57]. Altered levels of COX-2 have been implicated in cancers such as prostate, breast lung, and colorectal cancers, as well as OS. COX-2 expression was associated with low metastasis-free survival in patients with OS [58]. In another study in patients with OS lung metastases, it was found that COX-2 expression correlated inversely with disease-specific survival in these patients [59]. COX-2 inhibitor Celecoxib inhibition of cell proliferation and induction of apoptosis of human OS cell lines further supports the role of Cox-2 in OS [60,61]. A meta-analysis [62] involving 765 patients from 14 studies analyzed the relationship between COX-2 and metastasis of the tumor, clinical stage, and 3-year overall survival. High expression of COX2 was associated with neoplasm metastasis and advanced clinical stage, suggesting COX2 may be a candidate biomarker for OS. A study by Chen et al. [63] determined the relationship between hypoxia-inducible factor 1 α (HIF-1α), APE1, VEGF and COX-2 protein expression in pre-chemotherapy biopsies and determined the prognostic values. HIF α was found to correlate with COX2, VEGF, and APE1 as determined by immunohistochemistry.

A study [64] using surface enhanced laser desorption/ionization-time of flight-mass spectrometry (SELDI-TOF-MS) for cancer biomarker discovery—due to its ability to resolve low mass proteins and high-throughput capability—detected highly significant six different potential biomarkers in the serum. Microarray investigation demonstrated that the expression of 653 genes was changed more than 2-fold in three OS cell lines. It also showed increased expression of 310 genes and decreased expression in another 343 genes. Link test statistical analysis led to the identification of 13 genes which were potential biomarkers for the early diagnosis of OS. Among these, cytochrome c1 (CYC-1) was further analyzed. Western blot analysis was used to compare the expression of CYC-1 in OS patient samples and also to look at the levels of the protein before and after surgery. CYC1 was found to be higher in OS patients when compared to healthy controls. Interestingly CYC1 levels were significantly reduced in the same patients after surgery. These results suggest that CYC1 levels can be used as potential biomarkers for OS. However, further proof- of -concept studies are needed to validate the use of CYC1 as a biomarker for OS.

Many studies have assessed the prognostic role of upregulated p53 in patients presenting with OS. The authors of review article [65] summarized the existing evidence about whether the presence of upregulated p53 was a biomarker of survival in patients with OS using a meta-analysis of relevant publications. Upregulated p53 in patients was associated with lower 3-year overall survival and was also associated with decreased 3-year disease-free survival. Cells destroy themselves when they cannot repair lethal or sub-lethal damage in a process called apoptosis. Patients with Li Fraumeni syndrome, which is associated with germline mutations of the P53 gene, have a high incidence of developing OS [66].

Another meta-analysis analyzing the expression of p53 and pathogenesis of OS revealed no significant association between p53 protein expression and age, gender, tumor grade or cancer metastasis [67]. However, p53 expression was found to be lower in osteogenic OS than in non-osteogenic OS.

Src protein also belongs to the group of promising biomarkers of OS development and progression. In OS, Src protein plays an important role in different cellular processes (e.g., cell survival, adhesion and migration) through intracellular signal transduction activity [68]. Src can be activated by numerous signaling pathways to become phospho-Src. In this form other target proteins can be phosphorylated [69]. Hu et al. analyzed the expression of Src in OS patients. Their findings showed that Src overexpression is associated with metastasis and points to a poor prognosis [70]. The next study using an immunohistochemical method, showed the importance of certain cellular localizations of Src in determination of the prognosis. They showed that high nuclear staining was predictive of a good prognosis, while strong cytoplasmic staining was positively correlated with a poor prognosis [71].

The Hedgehog protein has been found to have a pivotal function in many cellular events, including morphogenesis and cell growth as well as tumor formation and metastasis. It also regulates OS progression and effects the metastasis of OS into various organs [72]. Lo et al. showed that a high level of Hedgehog signaling is associated with progression of high-grade OS [73]. However, there are several conflicting reports about the prognostic and diagnostic value of Hedgehog signaling [74,75]. On the other hand, the therapeutic potential of Hedgehog signaling has also been reported. For instance, Hedgehog/Gli pathway inhibitors prevent OS formation and inhibiting Hedgehog signaling positively affects the efficacy of OS therapy [76]. Moreover, by suppressing the Hedgehog pathway it is possible to increase the inhibitory effect on the radioresistance of OS cells [77]. Taken together, influencing the Hedgehog pathway may be a new approach in the OS treatment.

## 5. Enzyme Markers

Lactate dehydrogenase (LDH) catalyzes the interconversion of pyruvate and lactate in the glycolytic pathway and is a prognostic biomarker for different types of cancers including prostate, lung, and pancreatic cancers. A meta-analysis study showed an association between high serum LDH levels and a lower overall survival rate in patients with OS [78] and it is an effective biomarker of prognosis. The LDHB subunit was found to be highly expressed in OS cell lines [79]. Conversely, knockdown of LDHB led to decreased proliferation, migration and invasion in these cell lines. Moreover, higher levels of LDHB mRNA were observed in human OS tissues with metastasis versus those without metastasis. In the same study, it was found that patients with recurrence of OS, or advanced stage OS, showed an increased LDHB. These results suggest the potential for LDHB as a prognostic marker in OS.

Elevated levels of serum alkaline phosphatase (ALP) have been observed in OS patients in several studies [80,81,82]. A meta-analysis study showed that high ALP levels were associated with poorer overall survival in OS patients [83]. Other studies looked at expressions of ALP as well as LDH. In one such study, serum levels of ALP and LDH were analyzed pre- and post-chemotherapy. Both enzymes were found to be higher pre-chemotherapy compared to post-chemotherapy [84]. No correlation was observed between these enzymes and the percentage of tumor necrosis in OS patients.

## 6. Cell Adhesion Related Markers

Cell membranes are connected physically and functionally by ezrin (also known as cytovillin or violin-2). Ezrin belongs to the ezrin/radixin/moesin (ERM) protein family which links the plasma membrane and cytoskeleton. It plays an important role in cell surface structure adhesion, migration, and organization [85,86]. Ezrin expression has been linked to tumor metastasis [87,88]. In OS, ezrin expression has been linked to metastasis of the lungs [89] and inhibition of ezrin led to the reduction of metastasis [90]. All high-grade tumor patients were found to have high expression of ezrin [91]. In another meta-analysis study, ezrin expression was found to be positively associated with recurrence and poor survival [92]. High expression of the *Ezrin* gene in circulating tumor cells was also found to correlate with distant metastases [93]. Thus, several studies point to the positive association between high levels of ezrin and OS, suggesting that ezrin may be an effective marker of prognosis in OS patients.

## 7. Conclusion and Future Direction

Many basic, translational and clinical studies have identified biomarkers associated with OS. Based on these studies, various biomarkers could potentially be used to monitor the progression or predict the prognosis of OS. However, very few markers can be reliably used by the cancer caregiver to confidently make a confirmatory diagnosis early on in the disease progression, especially in pediatric patients. Achieving success in translating knowledge about the biomarkers from benchside to bedside requires carefully defined preclinical studies and properly controlled randomized clinical studies.

Advances in research methodology and technology have helped in the management of OS. However, due to the heterogeneous and complex nature of this disease, the survival rates have barely improved in last three decades. The question becomes how do we get past this stagnant response of OS to current treatment? Where do we go from here? One hope is to develop better prognostic and diagnostic tools so that appropriate measures can be taken in a timely manner, especially in the case of a metastatic OS. Identifying non-invasive, reliable biomarkers for early diagnosis is one such step towards improving survival in OS. One could potentially use the lessons learned from the biomarkers in other cancer types and utilize this information in finding reliable biomarkers for OS. Additionally, multidisciplinary approaches including identifying genetic targets combined with new therapies, and developing computer models and high throughput screening methodologies for predicting patient response, are some of the other promising candidates and directions for managing OS.

## Figures and Tables

**Table 1:** Osteosarcoma (OS) staging system of the Musculoskeletal Tumor Society (MSTS).

Enneking MSTS Staging System of OS	
Stage I	Low-grade, no metastasis
IA	Intra compartmental
IB	Extra compartmental
Stage II	High-grade, no metastasis
IIA	Intra compartmental
IIB	Extra compartmental
Stage III	Low or High-grade, presence of metastasis

**Table 2:** Overview of promising biomarkers in OS.

Biomarker	Function	Manifestation
miR-195	Diagnostic marker	↓ level in serum in OS patients in comparison with healthy individuals
miR-21	Diagnostic marker	↑ level in serum in OS patients in comparison with healthy individuals
TGF-β	Diagnostic marker	↑ level in serum in OS patients in comparison with healthy individuals
MMP-9	Prognostic marker	↑ expression correlates with adverse prognosis
HIF-1	Prognostic marker	↑ expression correlates with adverse prognosis
APE1	Prognostic marker	↑ expression correlates with reduced survival time
COX2	Metastasis prediction	↑ expression correlates with neoplasm metastasis
Src	Prognostic marker/metastasis prediction	↑ expression correlates with neoplasm metastasis and points to a poor prognosis
Ezrin	Diagnostic and prognostic marker	↑ expression in high-grade OS patients and points to a poor prognosis

miR—microRNA; TGF-β—transforming growth factor β; MMP-9—matrix metallopeptidase 9; HIF-1—hypoxia-inducible transcription factor 1; APE1—apurinic/apyrimidinic endonuclease 1; COX2—cyclooxygenase-2; Src—Proto-oncogene tyrosine-protein kinase Src.
